# Role of IGF-1/IGF-1R in regulation of invasion in DU145 prostate cancer cells

**DOI:** 10.1186/1475-2867-8-10

**Published:** 2008-07-03

**Authors:** Zeina Saikali, Hemani Setya, Gurmit Singh, Sujata Persad

**Affiliations:** 1 Department of Research, Juravinski Cancer Centre, Hamilton, Canada; 2 Department of Biochemistry and Biomedical Sciences, McMaster University, Hamilton, Canada; 3 Department of Pathology and Molecular Medicine, McMaster University, Hamilton, Canada; 4 Presently at: Department of Pediatrics, University of Alberta, Edmonton, Canada

## Abstract

**Background:**

Prostate cancer progression to androgen independence is the primary cause of mortality by this tumor type. The IGF-1/IGF-1R axis is well known to contribute to prostate cancer initiation, but its contribution to invasiveness and the downstream signalling mechanisms that are involved are unclear at present.

**Results:**

We examined the invasive response of androgen independent DU145 prostate carcinoma cells to IGF-1 stimulation using Matrigel assays. We then examined the signaling mechanisms and protease activities that are associated with this response. IGF-1 significantly increased the invasive capacity of DU145 cells *in vitro*, and this increase was inhibited by blocking IGF-1R. We further demonstrated that specific inhibitors of the MAPK and PI3-K pathways decrease IGF-1-mediated invasion. To determine potential molecular mechanisms for this change in invasive capacity, we examined changes in expression and activity of matrix metalloproteinases. We observed that IGF-1 increases the enzymatic activity of MMP-2 and MMP-9 in DU145 cells. These changes in activity are due to differences in expression in the case of MMP-9 but not in the case of MMP-2. This observation is corroborated by the fact that correlated changes of expression in a regulator of MMP-2, TIMP-2, were also seen.

**Conclusion:**

This work identifies a specific effect of IGF-1 on the invasive capacity of DU145 prostate cancer cells, and furthermore delineates mechanisms that contribute to this effect.

## Background

Insulin-like growth factor 1 (IGF-1), via binding to the IGF-1 receptor (IGF-1R), is thought to contribute to the development of prostate cancer by promoting proliferation and blocking apoptosis [1,2], which likely account for the epidemiological findings of association between IGF-1 or elements of its regulatory system and the development of prostate cancer [3]. The role of IGF-1 in the progression of prostate cancer to an invasive and metastatic phenotype is still unclear, although it has been studied in other tumour types. Increased IGF-1R signalling is associated with an upregulation of extracellular proteases necessary for tumour cell invasion in lung and breast cancer [4], and suppression of IGF-1R in breast cancer decreases tumour metastasis *in vivo *[5]. The association between IGF-1R and prostate cancer progression is less clear. There is clinical data showing lack of correlation between IGF-1 levels and stage of disease [6,7], yet there is also evidence of significantly increased IGF-1R expression in advanced disease [8]. Furthermore, data from an animal model of prostate cancer progression and a prostate cancer cell line indicate an effect of IGF-1R signalling on invasion [9,10]. This suggestive data, however, does not establish a direct causative role for IGF-1 signalling in the promotion of prostate cancer progression to an invasive phenotype. IGF-1/IGF-1R activates a number of signalling pathways, including the phosphatidylinositol-3 kinase (PI3-K) pathway, the protein kinase C pathway, the CREB pathway and the mitogen activated protein kinase (MAPK) pathway [11-14], but the relative contribution of these pathways in prostate cancer cell invasion is unknown. Prostate cancer often exhibits inactivation of a major regulator of the PI3-K pathway, PTEN, leading to deregulation and constitutive activation of this pathway. Thus, the contribution of these two pathways to IGF-1-stimulated invasion of prostate cells requires further analysis. In order to do this, we studied IGF-1-stimulated invasion in the DU145 cell line, which is the only commercially available prostate cancer cell line without PTEN inactivating mutations and an intact, tightly regulated PI-3 kinase pathway[15-17]. Our study specifically determined that IGF-1/IGF-1R signaling via the PI3-K and MAPK pathways augments the invasive phenotype of these prostate cancer cells, and that this regulation is at least partially attributed to an increase in the activity, but not necessarily in the expression, of MMP-2 and MMP-9.

## Methods

### Cell culture and Matrigel invasion assay

The DU145 cell line, obtained from the American Type Culture Collection (Manassas, VA), was cultured in Dulbecco's modified eagle's medium (DMEM; Sigma-Aldrich Canada Ltd., Oakville, ON) supplemented with 10% fetal bovine serum (FBS; HyClone, Logan, UT), 50 μg/ml penicillin G sodium and 50 μg/ml streptomycin sulfate (Invitrogen Canada Inc., Burlington, ON). IGF-1 was obtained lyophilized from Sigma-Aldrich and reconstituted in distilled water. Fifty thousand DU145 cells were added per invasion chamber coated with Matrigel (reconstituted basement membrane; BD Biosciences, Mississauga, ON). Cells were allowed to invade for 24 hours towards media containing 10% FBS and the number of invaded cells were counted according to the manufacturer's instructions. Where indicated, one of three inhibitors were used: 100 nM wortmannin (Sigma-Aldrich), a concentration chosen from a range used in the literature[18-20]; 50 μM PD98059 (Sigma-Aldrich), a concentration chosen from a range used in the literature[18,21,22]; or 1 μg/mL of an IGF-1R neutralizing antibody, MAB391 (R&D Systems, Inc., Minneapolis, MN), a concentration equivalent to about 6 nM, found to be effective in significantly reducing IGF-1R phosphorylation[23].

### Preparation of cell lysates and conditioned media

Washed cell pellets were lysed in 1% NP-40, 150 mM NaCl, 50 mM Tris pH7.6, 1 mM EDTA containing 10% protease inhibitor cocktail (Roche Diagnostics, Laval, QC) and kept on ice for 1 hour with intermittent vortexing. Extracts were centrifuged at 1000 rpm for 5 minutes at 4°C and the supernatant was collected. Protein levels were quantified using the Bradford assay (Bio-Rad Laboratories, Mississauga, ON). Conditioned media was centrifuged at 1000 rpm for 5 minutes at 4°C to eliminate cellular debris. Cell number was determined for each sample. The media was concentrated using Amicon Ultra-4 centrifugal filter units (Millipore Canada, Etobicoke, ON) with a molecular weight cut-off of 10 kD, as per the manufacturer's recommendations.

### Immunoblot analysis

Proteins were separated by SDS-PAGE and transferred to a PVDF membrane (Bio-Rad). Membranes were probed with the relevant primary antibodies: mouse anti-MMP-2 monoclonal Ab (1:100, Oncogene Research Products, La Jolla, CA); mouse anti-MMP-9 monoclonal Ab (1:200, R&D Systems); goat anti-MMP-9 polyclonal Ab (1:1000, Santa Cruz Biotechnology, Inc., Santa Cruz, CA); mouse anti-P-Akt monoclonal Ab (1:1000, Cell Signaling Technology, Inc., Danvers, MA); rabbit anti-P-MAPK polyclonal Ab (1:1000, Cell Signaling); mouse anti-TIMP-1 monoclonal Ab (1:100, Cedarlane Laboratories, Hornby, ON); mouse anti-TIMP-2 monoclonal Ab (1:400, Cedarlane Laboratories) and mouse anti-actin monoclonal Ab (1:10 000, MP Biomedicals, Inc., Aurora, OH). Secondary antibodies were all horseradish peroxidase conjugated [goat anti-mouse IgG (Bio-Rad); goat anti-rabbit IgG (Bio-Rad); donkey anti-goat IgG (Bio-Rad), all at 1:10000]. Visualization of antibody binding was carried out using enhanced chemiluminescence (ECL; Perkin Elmer Life Sciences, Woodbridge, ON) and exposure to Kodak X-OMAT film.

### Gelatin zymography

Conditioned media was prepared in sample buffer (0.25 M Tris-HCl pH6.8, 10% SDS, 4% sucrose, 0.1% bromophenol blue) and electrophoresed on 8% polyacrylamide gels containing 0.3% gelatin. The gels were washed with 2.5% Triton X in dH_2_O and incubated for 48 hours at 37°C in substrate buffer (50 mM Tris-HCl, 0.2 M NaCl, 5 mM CaCl_2_, 0.02% Brij35). After incubation, the gels were stained with Coomassie Blue solution (10% glacial acetic acid, 30% isopropanol, 0.5% Coomassie Blue R250), destained (40% methanol, 10% acetic acid, 50% water) until clear bands representing zymogen activity appeared, then dried.

## Results

### IGF-1 increases the invasive potential of DU145 prostate cancer cells through the IGF-1R, via both the PI3-K and MAPK pathways

**Figure 1:**
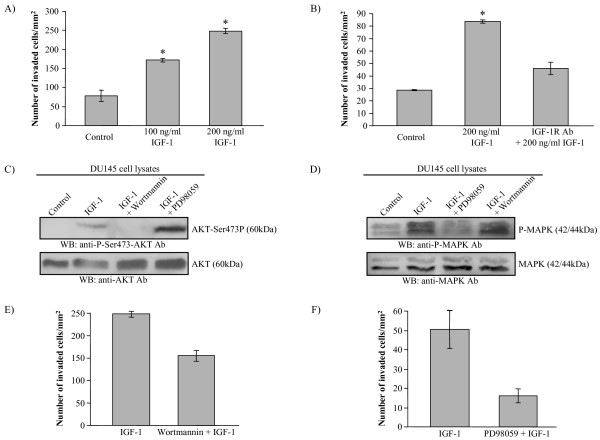
**IGF-1 stimulates the *in vitro *invasion of DU145 cells through the IGF-1R, via both the PI3-K and MAPK pathways**. **A) **Representative experiment showing number of cells invading a Matrigel-coated membrane relative to surface area. Serum-deprived DU145 cells were treated for 24 hours with indicated concentrations of IGF-1 after which 5 × 10^4 ^cells were allowed to invade through the Matrigel for 24 hours. IGF-1 treatment induces a dose-responsive increase in the invasive potential of DU145 cells through Matrigel compared to invasion in mock-treated cells that were administered a volume of 1× PBS similar to the 200 ng/ml condition. (*p < 0.05; Fisher exact T-test). **B) **Number of cells invading a Matrigel-coated membrane relative to surface area. Serum-deprived DU145 cells were pretreated for 24 hours with a neutralizing IGF-1R antibody (IGF-1Rab), then treated with 200 ng/ml IGF-1 for 24 hours. 5 × 10^4 ^cells were allowed to invade through the Matrigel for 24 hours. The increase in Matrigel invasion of DU145 cells stimulated by IGF-1 is significantly decreased in the presence of the IGF-1R neutralizing antibody (*p < 0.05; Fisher exact T-test) The control consisted of adding a similar amount of the vehicle (1× PBS) for each addition in the test conditions. **C,D,E,F) **Serum-deprived DU145 cells were pre-treated in the presence or absence of the PI3-K inhibitor wortmannin or the MEK inhibitor PD98059 for 1 hour, then treated with 200 ng/ml IGF-1 for 1 hour, maintaining previous inhibitor conditions. The control consisted of adding a similar amount of the vehicle (1× PBS for IGF-1, DMSO for wortmannin or PD98059) for each addition in the test conditions. **C) **Immunoblotting shows an increase in Akt phosphorylation in DU145 cells treated with IGF-1, but not in the presence of wortmannin. **D) **Immunoblotting shows an increase in the phosphorylation of p42/44 MAPK in DU145 cells treated with IGF-1, but not in the presence of PD98059. **E,F) **Representative experiments showing number of cells invading a Matrigel-coated membrane relative to surface area. At the end of the respective treatments, 5 × 10^4 ^cells were added to each invasion chamber and allowed to invade through the Matrigel for 24 hours. **E) **Each condition was performed on three separate occasions. The relative rate of inhibition by wortmannin of the IGF-1 effect on invasion was consistent across experiments, with a mean of 0.79 +/- 0.083. **F) **Each condition was performed on four separate occasions. The relative rate of inhibition by PD98059 of the IGF-1 effect on invasion was consistent across experiments, with a mean of 0.37 +/- 0.096.

The effect of IGF-1 on the in vitro invasive potential of DU145 cells was investigated using Matrigel invasion assays. Figure 1A shows that IGF-1 treatment results in a dose-responsive increase in the invasive potential of DU145 cells compared to untreated cells. When DU145 cells were treated with an IGF-1R neutralizing antibody that competes with IGF-1 binding to IGF-1R and induces receptor degradation [23], the IGF-1-induced increase in invasion of DU145 cells was attenuated to close to baseline values (Fig. 1B). This indicates that the observed invasive phenotype of DU145 cells is due specifically to IGF-1 signalling through its receptor. To study the effects of IGF-1 via the PI3-K pathway, P-Akt levels were assessed and found to be upregulated in DU145 cells following 1-hour IGF-1 treatment (Fig. 1C). This stimulation is inhibited by wortmannin, a selective, irreversible inhibitor of the PI3-K pathway, but not by PD98059, a potent inhibitor of the MAPK pathway. We next evaluated the activation of the MAPK pathway by IGF-1 by determining increases in phosphorylated p42/44 MAPK (p42/44 P-MAPK). We noted an increase in p42/44 P-MAPK with IGF-1 stimulation that decreased to baseline levels in the presence of PD98059 but not wortmannin (Fig. 1D). The increased invasion of DU145 cells induced by IGF-1 was significantly inhibited in the presence of either wortmannin (by a ratio of 0.79 +/- 0.083) or PD98059 (by a ratio of 0.37 +/- 0.096) (Fig. 1E&1F). This data indicates a regulatory role of IGF-1 signalling in invasion via both the PI3-K and MAPK pathways.

### IGF-1 regulates MMP-2 and MMP-9 activity and expression via the PI3-K and MAPK pathways

**Figure 2:**
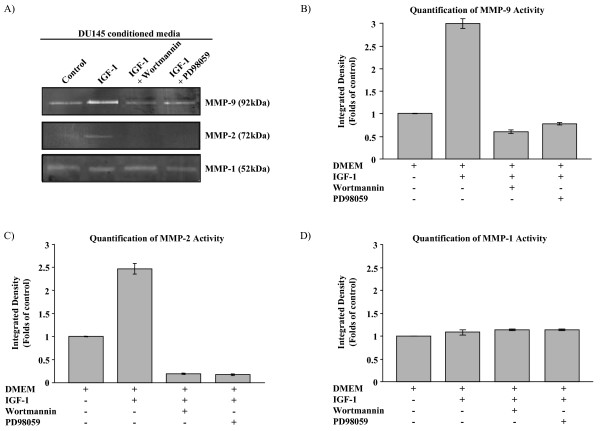
**IGF-1 induces the activity of MMP-9 and MMP-2 via both PI3-K and MAPK pathways**. Serum-deprived DU145 cells were pre-treated for 1 hour with either the PI3-K inhibitor wortmannin or the MEK inhibitor PD98059, then treated with IGF-1 for 24 hours in the same conditions of inhibitor use. The control consisted of adding a similar amount of the vehicle (1× PBS for IGF-1, DMSO for wortmannin or PD98059) for each addition in the test conditions. Conditioned media was concentrated, normalized to cell number and used for gelatin zymography. Protease activity is seen as clear digested bands at 92 kDa for MMP-9, at 72 kDa for MMP-2 and at 52 kDa for MMP-1 **(A)**. Densitometric quantification indicates that MMP-9 activity is increased by IGF-1, with this increase prevented by either wortmannin or PD98059 **(B)**. MMP-2 activity is also induced by IGF-1; presence of either inhibitor completely abrogates this activation **(C)**. The activity of MMP-1 is unaffected by IGF-1 both in the absence and presence of wortmannin or PD98059 **(D)**.

**Figure 3:**
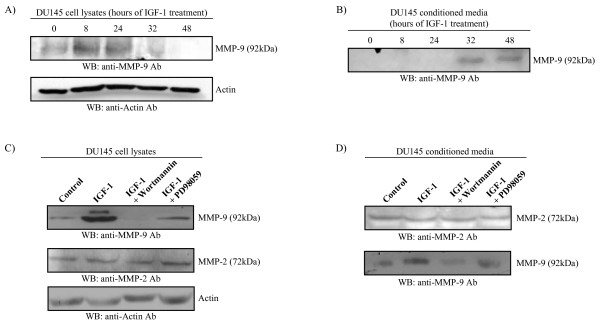
**IGF-1 regulates the activity of MMP-9 and MMP-2 by different mechanisms**. **A, B) **DU145 cells were treated with IGF-1 for varying amounts of time and both cell lysate and conditioned media were collected. **A) **Immunoblot of cell lysates indicates that IGF-1 induces an increase in the cellular protein expression of MMP-9 in a time-dependent manner at 8 and 24 hours of treatment, then a decrease at 32 and 48 hours of treatment. **B) **Immunoblot of conditioned media indicates that IGF-1 induces an increase in the secreted protein expression of MMP-9 in a time-dependent manner seen after 32 hours of treatment. The amount of protein in each lane is normalized to cell number. **C, D)**DU145 cells were pre-treated with the PI3-K inhibitor wortmannin or the MEK inhibitor PD98059, then treated with IGF-1 for 8 hours and both cell lysate and conditioned media were collected. The control consisted of adding a similar amount of the vehicle (1× PBS for IGF-1, DMSO for wortmannin or PD98059) for each addition in the test conditions. **C) **Immunoblot of lysates indicates that the IGF-1-induced increase in the cellular protein expression of MMP-9 is attenuated in the presence of PD98059 and even more so in the presence of wortmannin. No change in MMP-2 expression is seen in any of the conditions. **D) **Protein expression of MMP-2 in conditioned media is unaltered compared to mock-treated control cells, whereas that of MMP-9 follows the same pattern seen in cell lysates. The amount of protein in each lane is normalized to cell number. All blots are representative of three separate experiments in which a similar trend was observed.

MMPs have been identified as being highly associated with prostate cancer invasion [24]. Gelatin zymography, analyzing the ability of MMPs to degrade gelatin, was performed to identify possible alterations in MMP activity due to IGF-1 stimulation. Following 24-hour treatment of DU145 cells with IGF-1, MMP-9 and MMP-2 activity was increased and this enhanced activity was inhibited or abolished in the presence of wortmannin or PD98059 (Fig. 2). There was no change in MMP-1 expression after IGF-1 treatment (Fig. 2), indicating that the activity of this protein does not appear to be regulated by IGF-1, and that the effects of IGF-1 are specific for certain MMPs. Intracellular and secreted protein levels were examined using immunoblot analysis of cell lysates and conditioned media, respectively. IGF-1 initially induced an increase in MMP-9 intracellular protein expression with time (8 hrs and 24 hrs), followed by a decrease at longer time points (Fig. 3A). Extracellular protein expression of MMP-9 was observed at 32 hrs and 48 hrs of IGF-1-treatment (Fig. 3B), indicating secretion of MMP-9 due to stimulation with IGF-1. The increase in cellular expression of MMP-9 with 8-hour IGF-1 treatment was found to be attenuated in the presence of either of the inhibitors wortmannin or PD98059 (Fig. 3C). On the other hand, MMP-2 intracellular protein expression did not change with IGF-1 treatment over the course of 48 hours (results not shown), irrespective of the presence of wortmannin and PD98059 (Fig. 3C). Similarly, secreted levels of MMP-2 also showed no change with 24-hour IGF-1 treatment (Fig. 3D).

### IGF-1 regulation of TIMP-2 secreted protein levels via the PI3-K and MAPK pathways

**Figure 4:**
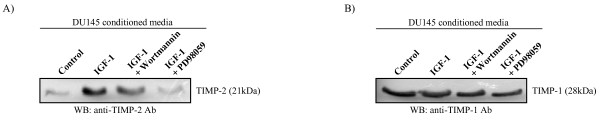
**IGF-1 regulates secreted TIMP levels**. DU145 cells were pre-treated for 1 hour with either the PI3-K inhibitor wortmannin or the MEK inhibitor PD98059, then treated with IGF-1 for 24 hours in the same conditions of inhibitor use. The control consisted of adding a similar amount of the vehicle (1× PBS for IGF-1, DMSO for wortmannin or PD98059) for each addition in the test conditions. Conditioned media from these treatments were concentrated and probed with (**A) **anti-TIMP-2 antibody or (**B**) anti-TIMP-1 antibody. The amount of protein in each lane is normalized to cell number. Blots are representative of three separate experiments in which a similar trend was observed.

Since we did not observe any alterations in protein expression of MMP-2, it is likely that IGF-1 regulates MMP-2 activity by mechanisms other than an increase in its cellular expression. TIMP-1 and TIMP-2 are inhibitors of MMPs and have been shown to be involved in their regulation. Specifically, TIMP-1 has a negative regulatory effect on MMP-9 and TIMP-2 is known to have a dual stimulatory and inhibitory effect on the activity of on MMP-2 [25]. Therefore, we looked for alterations in the secreted protein levels of TIMP-1 and TIMP-2 upon IGF-1 treatment. Secreted TIMP-2 levels were found to be increased in the presence of IGF-1 and decreased in the presence of IGF-1 and wortmannin or PD98059 (Fig. 4A). Therefore, TIMP-2 levels are regulated by IGF-1 via the PI3-K and MAPK pathways, paralleling the regulation of the activity of MMP-2 by IGF-1 as determined by gelatin zymography (Fig. 2). On the other hand, secreted TIMP-1 levels remain unchanged in the presence of IGF-1 (Fig. 4B).

## Discussion

Studies described herein have addressed whether IGF-1 has a direct effect on the invasive potential of DU145 prostate carcinoma cells and have established some of the mechanisms involved. This is the first study, to our knowledge, showing that the addition of exogenous IGF-1 to prostate cancer cells results in a significant increase in invasive potential, and that these effects are reduced by inhibiting IGF-1R, the MAPK pathway or the PI3-K pathway. Previous studies have shown that the inhibition of IGR-1R reduced invasion in the PC-3 prostate cancer cell line [10], confirming the requirement for IGF signalling but not showing that it was sufficient alone to induce invasion. Thus, surprisingly, a positive effect of IGF-1 alone on invasive capacity in this tumour type had not been documented until now. The inhibition of IGF-1R phosphorylation using the MAB391 neutralizing antibody is dose-dependent in DU145 cells and the observed inhibition can be reversed in the presence of excess IGF-1 [23], suggesting that MAB391 inhibits IGF-1R phosphorylation in a manner that is competitive with the IGF-1 ligand.

The data indicates a regulatory role of IGF-1 signalling via both the PI3-K and MAPK pathways in DU145 prostate carcinoma cells. Phosphorylation of key elements of these pathways, Akt and MAPK, following IGF-1 treatment confirmed the role of the PI3-K and MAPK pathways in IGF-1 signalling and correlates with invasive capacity. P-Akt levels were found to be decreased in the presence of wortmannin as expected; however they also increased in the presence of PD98059, possibly due to increased IGF-1 signalling through the PI3-K pathway when the MAPK pathway is blocked. Contrary to the results described here, Pfeil and colleagues [26] showed that MEK inhibition did not affect P-Akt activation in DU145 cells. This difference using the same cell line could be due the use of a lower drug concentration of 20 μM, perhaps insufficient to effectively block the MAPK pathway, whereas our cells were treated with 50 μM PD98059. Our results also show that IGF-1-induced P-MAPK levels were decreased by PD98059 as expected, and unaffected by wortmannin. Together, these results indicate that IGF-1/IGF-1R signalling via the PI3-K and MAPK pathways leads to enhanced invasive capacity in DU145 cells, and that inhibition of either pathway impairs invasion.

We examined the effects of IGF-1 on the collagenolytic activity of DU145 cells using gelatin zymography which is an extremely sensitive technique that can detect picogram levels of MMPs. There is precedence for the role of IGF-1 in this regard via its effects on MMPs, such as MMP-2 and MMP-9 in MCF-7 breast cancer cells and in androgen-independent PC3 prostate cancer cells [27,10]. IGF-1 was shown to increase the activity of MMP-2 and MMP-9; however MMP-1 levels remained unchanged, indicating specificity of action of IGF-1. Both MMP-2 and MMP-9 activity levels were decreased in the presence of either wortmannin or PD98059, indicating that the regulatory role of IGF-1 on enzyme activity is transmitted via the PI3-K and MAPK pathways. Inhibition of either signalling pathway resulted in complete inhibition of MMP-2 activity, suggesting the requirement of activation of both pathways in MMP-2 regulation. On the other hand, MMP-9 activity was decreased to baseline levels in the presence of either wortmannin or PD98059. The increase in activity of MMP-9 induced by IGF-1 was found to correlate with an increase in protein expression and secretion from the cell, whereas this was not the case for MMP-2 (Fig. 3).

It is the balance between MMPs and their inhibitors (TIMPs) that determines the proteolytic degradation of the matrix and if this balance is disrupted, prostate tumour growth and progression are significantly affected [28]. We analyzed the response of both TIMP-1 and TIMP-2 to IGF-1 and found that TIMP-1 levels are unaltered, suggesting that TIMP-1 expression is not regulated by this growth factor. On the other hand, secreted TIMP-2 levels increased with IGF-1 and decreased in the presence of wortmannin and PD98059, paralleling the results seen for MMP-2 and MMP-9 enzyme activity. Therefore, IGF-1 appears to have a stimulatory effect on TIMP-2 protein expression, and on MMP-2 and MMP-9 activity. TIMP-2 is known to bind to proMMP-2 and facilitate enzyme activation [29]. The correlation between TIMP-2 protein levels and MMP-2 activity indicates that TIMP-2 induction may be a possible mechanism of stimulation of MMP-2 activity by IGF-1. To verify this however, further experiments would be needed, such as the analysis of IGF-1-stimulated MMP-2 activity in DU145 cells in the presence of TIMP-2 inhibition.

## Conclusion

The results from this study show that IGF-1 is a key regulator of the invasive potential of DU145 prostate cancer cells. This effect of IGF-1 appears to be mediated at least in part through its ability to regulate MMP-2 and MMP-9 activity and secreted TIMP-2 protein levels, effects which are transduced via the PI3-K and MAPK pathways. Further work is necessary to elucidate additional elements of the complete mechanism for IGF-1 induction of prostate cancer invasion.

## List of abbreviations used

IGF: Insulin-like growth factor; MMP: Matrix metalloproteinase; PI3-K: Phosphatidylinositol-3 kinase; MAPK: Mitogen activated protein kinase; FBS: Fetal bovine serum.

## Competing interests

The authors declare that they have no competing interests.

## Authors' contributions

ZS carried out the invasion assays, statistical analysis and drafted the manuscript, HS carried out the zymograms and protein expression studies and helped with the statistical analysis, GS revised the manuscript critically for important intellectual content, SP conceived of the study, designed and coordinated the study and helped draft the manuscript, SP also gave final approval for the paper to be submitted for publication.
